# Antagonistic Effects of Enrofloxacin on Carbendazim-Induced Developmental Toxicity in Zebrafish Embryos

**DOI:** 10.3390/toxics9120349

**Published:** 2021-12-10

**Authors:** Ruiqi Fan, Wanjun Zhang, Li Jia, Sunlin Luo, Ying Liu, Yongpeng Jin, Yongchen Li, Xiaoyan Yuan, Yiqiang Chen

**Affiliations:** 1 State Key Laboratory of Animal Nutrition, College of Animal Science and Technology, China Agricultural University, Beijing 100193, China; fanruiqi94110@cau.edu.cn (R.F.); wanjunzhang@cau.edu.cn (W.Z.); lsl18810791522@163.com (S.L.); liuyingcau2021@163.com (Y.L.); jinyp@cau.edu.cn (Y.J.); liyongc2021@163.com (Y.L.); 2 Center of Disease Control and Prevention, PLA, Beijing 100073, China; jiali1230@aliyun.com (L.J.); yanziyuan2007@126.com (X.Y.); 3 School of Nursing and Health, Henan University, Kaifeng 475000, China

**Keywords:** carbendazim, enrofloxacin, zebrafish, embryonic developmental toxicity, antagonism

## Abstract

Carbendazim (CAR) and enrofloxacin (ENF) are frequently detected in fruits and meat products, respectively. Since most people consume fruits, vegetables, and meat products, combined exposure is possible, necessitating further evaluation of toxic interactions. In this study, the developmental toxicity of separate and combined exposure was examined in zebrafish embryos. Carbendazim exposure at 0.79 mg/L and above significantly affected developmental parameters, while enrofloxacin alone had no substantial effects on these developmental parameters within the selected concentration range (0.10–0.40 mg/L). Surprisingly, ENF antagonized the CAR-evoked reduction in the 48 hpf (hours post-fertilization) hatching rate and the increases in the 96 hpf malformation and lethality rates. The results revealed that the antagonism might be associated with reciprocal effects of these compounds on metabolism-related genes, such as *cyp7a1* and *apoa1a*. These results reveal a complex interaction between ENF and CAR on metabolic regulation during development and highlight the importance of combined assessment for agents with the potential for simultaneous exposure.

## 1. Introduction

Enrofloxacin (ENF) is a fluoroquinolone antibiotic widely used in animal production and generally considered safe for livestock and of low exposure risk to agricultural workers. However, recent reports indicate that long-term use may result in relatively high residual levels in the eggs of treated hens [1,2]. Further, ENF treatment of mares was reported to damage foetal cartilage [3]. Additionally, low doses of ENF to mice can cause changes in the intestinal flora and induce bacterial resistance [4]. Collectively, these studies indicate that the risks of ENF to human health warrant further evaluation.

Carbendazim (CAR) is a broad-spectrum benzimidazole fungicide prohibited in the United States due to substantial toxicity. However, it is still used on a large scale in several other countries, and there are reports of residual levels in excess of allowable limits on various fruits and vegetables grown in China [5]. Studies have confirmed that CAR can interfere with endocrine function [6] and reproductive function [7] in rats.

In modern agricultural practice, multiple pesticides, fungicides, and antibiotics may be used to protect crops and livestock, increasing the risk of simultaneous exposure and unexpected toxic interactions. Most reports on mixed toxicity concern multiple pesticides [8,9] or antibiotics [10,11], while there has been relatively little study on the interactions between crop pesticides and veterinary antibiotics. CAR is one of the most frequently detected pesticides in China [12], and ENF is on the list of the most frequently detected veterinary antibiotics in China [13]. Given that most people consume animal products, fruits, and vegetables daily, a comprehensive human health risk assessment should include cross-category toxicity. Moreover, the cross-category toxicity of CAR and ENF still lacks investigation, and it is necessary to explore the combined toxicity of the two chemicals.

Zebrafish share a high degree of genetic homology with humans and have the advantage of integrating in vitro cells and in vivo animal models [14]. The zebrafish model is of high efficiency and low cost. In recent years, it has been widely used in the field of drug research and development for the toxicity evaluation of compounds and the high-throughput screening of active compounds. Thus, zebrafish embryos were selected as the model of our research [15].

Large-scale gene expression profiling by transcriptomics can help reveal the molecular mechanisms of joint toxicity [16,17]. In this study, the individual and combined effects of CAR and ENF were examined on embryonic zebrafish development, followed by transcriptomics with qPCR verification of gene expression changes for preliminary examination of the underlying molecular mechanisms. We intended to explore the complex interaction between ENF and CAR on metabolic regulation during development, and highlight the importance of combined assessment for agents with the potential for simultaneous exposure.

## 2. Materials and Methods

### 2.1. Reagents

Carbendazim (99%) was purchased from MedChemExpress (Monmouth Junction, NJ, USA), enrofloxacin (>98%) from Sigma-Aldrich (Saint Louis, MO, USA), and DMSO (99.7%) from Thermo Fisher (Waltham, MA, USA). All other reagents were of analytical grade and purchased from Shanghai Sinopharm Group Chemical Reagents.

### 2.2. Zebrafish Breeding and Egg Collection

Wild-type Tu zebrafish were purchased from Wuhan Zebrafish Resource Center (Wuhan, China) and maintained in a dedicated breeding system from ESEN Technology (Beijing, China) under a 14 h/10 h light cycle and ambient room temperature of 28 ± 2 °C. Animals were fed freshly hatched brine shrimp larvae twice daily. In the afternoon before experiments, two male and two female fish were placed in separate compartments of a breeding box. At the beginning of the photoperiod the following day, the partition separating compartments was removed to allow the males and females to chase freely. After mating, the fertilized eggs were collected within 30 min.

### 2.3. Zebrafish Embryo Exposure and Developmental Index Detection

Pesticides were weighed, dissolved in DMSO as stock solutions, and diluted in Holt buffer (containing NaCl 3.5 g/L, KCl 0.05 g/L, CaCl_2_ 0.1 g/L, NaHCO_3_ 0.05 g/L) to the indicated treatment concentrations. The final DMSO concentration (0.1%) had no detectable effects on zebrafish development in published papers [18,19]. The final ENF concentrations were 0.10, 0.13, 0.16, 0.20, 0.26, 0.32, and 0.40 mg/L, and the final CAR concentration were 0.50, 0.63, 0.79, 1.00, 1.26, 1.59, and 2.00 mg/L. The concentrations selected were based on the maximum residual levels of CAR (0.05–3 mg/kg [20]) and ENF (0.1–0.3 mg/kg [21]) in food in China. The mixed exposure liquid (MIX) (ENF: CAR = 1:4.84, *v*/*v*) was prepared according to the acceptable daily intake (ADI) from the Ministry of Agriculture and Rural Affairs of the People’s Republic of China [20,21]. Total MIX concentrations were 0.54, 0.68, 0.86, 1.09, 1.37, 1.72, and 2.17 mg/L. The CAR concentrations in MIX were 0.09, 0.12, 0.15, 0.19, 0.23, 0.29, and 0.37mg/L, and the ENF concentrations in MIX were 0.45, 0.56, 0.71, 0.90, 1.14, 1.43, and 1.80 mg/L. 

Eggs were collected at 4 hpf (hours post-fertilization) and examined under an SZ-10 stereomicroscope (Olympus, Japan) to remove unfertilized, coagulated, and damaged samples. Twenty healthy eggs were placed in each well of a 6-well plate and treated with 10 mL of the indicated solution, with three replicates at each concentration. The solution was exchanged every 24 h for 96 h and dead eggs were removed at each exchange. Embryonic hatching, mortality, and malformation rates of each treatment group were recorded at 24, 48, 72, and 96 hpf.

### 2.4. Exposure for Transcriptomics

Exposure concentrations for transcriptomics were set according to the benchmark doses (BMDL_10_ values) of ENF alone, CAR alone, and the mixture. Fertilized eggs were treated from 4 hpf to 96 hpf, with three biological replicates per treatment group and 80 eggs per replicate. After 96 hpf, treated eggs in each group were transferred to a 2 mL centrifuge tube, and the supernatant was discarded by centrifugation at 12,000 rcf and 4 °C for 10 min. Larvae were then washed twice with 1 mL PBS, with centrifugation and supernatant removal between washes. RNA was extracted using Trizol reagent according to the manufacturer’s instructions (Ambion, Austin, TX, USA) and stored at −80 °C for further testing. 

### 2.5. mRNA Library Construction

RIN (RNA integrity number) and concentration were assessed using Fragment Analyzer 5400 (Agilent, Santa Clara, CA, USA), and the RINs were all above 9. Oligo(dT)-attached magnetic beads were used to purify mRNA. The First-strand cDNA was then generated through a random hexamer-primed reverse transcription, followed by second-strand cDNA synthesis. The single-strand circle DNA (ssCir DNA) was formatted as the final library. The final library was amplified with phi29 to make DNA nanoballs (DNB), with more than 300 copies of one molecular. DNBs were loaded into the patterned nanoarray, and single-end 50 bases reads were generated on the BGIseq500 platform (BGI-Shenzhen, China). The transcriptome raw data were submitted to the NCBI database with the BioProject number PRJNA780808.

### 2.6. RNA-Seq Data Analyze

The filter of the sequencing data was with SOAPnuke [22]. Low-quality and unknown reads were removed. Then, clean reads were obtained and stored in FASTQ format. HISAT2 (v2.0.4) was used to map the clean reads to the reference genome [23]. Bowtie2 (v2.2.5) was applied to align the clean reads to the reference coding gene set [24]. The expression level of genes was calculated by RSEM (v1.2.12) [25]. The reference database was from NCBI (GRCz11). Essentially, differential expression analysis was performed using the DESeq2(v1.4.5) [26]. GO (http://www.geneontology.org/, accessed on 6 July 2021) and KEGG (https://www.kegg.jp/, accessed on 7 July 2021) enrichment analysis of annotated different expressed genes was then performed to explore the biological function further. The significant terms and pathways were corrected by a *p*-value with a rigorous threshold (*p*-value ≤ 0.05). The heatmap and GO network were completed through Hiplot (https://hiplot.com.cn/, accessed on 5 October 2021). 

### 2.7. Real-Time Quantitative Reverse Transcription PCR 

To verify BGI-Seq results, RNA extracted as described was reverse transcribed into cDNA using a reverse transcription kit (Thermo Scientific, Waltham, MA, USA) with SYBR and the primer sequences listed in Supplementary Materials on an LightCycler480 II thermocycler (an LightCycler480 II thermocycler) instrument. The reaction volume was 25 μL and the thermocycle program was 95 °C for 3 min, 45 cycles of 95 °C for 5 s, 60 °C for 30 s, and dissociated according to instrument guidelines. Expression levels relative to β-actin as the reference gene were calculated using the 2^−ΔΔCT^ method (primer pairs of selected genes can be found in Table S1).

### 2.8. Data Analysis and Statistics

SPSS 22.0 was used for statistical analysis of all developmental parameters and to calculate the LC_50_ values. Developmental parameters and qPCR analysis results were expressed as mean ± standard deviation. One-way analysis of variance (ANOVA) and Tukey’s test were used to compare the means of multiple groups if they passed Levene’s Test. *p*-values < 0.05 were considered to be statistically significant. Graphpad 8.0 was used to draw 96 hpf dose-response curves and BMDS 3.2 software (EPA, Washington, DC, USA) to calculate BMDL_10_ values [27]. CompuSyn 1.0.4 software [28] was used to construct 96 hpf concentration—lethality curves and concentration—deformation rate curves, and to calculate joint toxicity effects by combination index (CI) analysis. Transcriptomic results were analyzed using the BGI Dr.Tom online work platform (https://biosys.bgi.com, accessed on 1 October 2021).

## 3. Results

### 3.1. Toxicity of CAR Alone on Zebrafish Embryo Development

**Figure 1:**
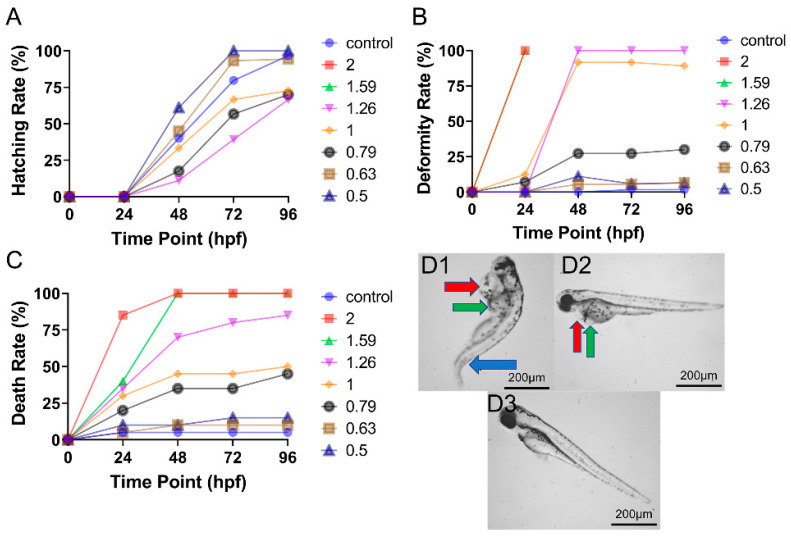
Concentration- and time-dependent effects of CAR on zebrafish embryo development (n = 60). (**A**) Time–response curves for hatching rate at all tested doses. (**B**) Time–response curves for deformity rate at all tested doses. (**C**) Time–response curves for death rate at all tested doses. (**D**) Typical deformities induced by (**D1**) 1.26 mg/L, (**D2**) 1.00 mg/L, and (**D3**) 0.50 mg/L CAR at 96 hpf. Red arrows demarcate areas of pericardial oedema, green arrows demarcate regions of yolk sac oedema, and blue arrows show regions of spinal curvature.

Carbendazim significantly altered the hatching, deformity, and lethality rates of zebrafish embryos (Figure 1). At 48 and 72 hpf, low-dose CAR (0.50–0.63 mg/L) increased the hatching rate of zebrafish embryos, while high-dose CAR (0.79–1.26 mg/L) decreased the hatching rate at 48 and 72 hpf (Figure 1A). Carbendazim also significantly and dose-dependently enhanced deformation and lethality rates (Figure 1B,C). High-dose CAR (1.00–2.00 mg/L) significantly increased the deformity rate from 0 to 48 hpf, reaching 90% by 48 hpf. The primary malformations caused by high-dose CAR included pericardial oedema, yolk sac oedema, and spinal curvature (Figure 1D1). High-dose CAR (1.59–2.00 mg/L) also dramatically enhanced embryonic lethality between 0 and 48 hpf, reaching nearly 100% at 48 hpf, while medium-dose CAR (0.79–1.26 mg/L) eliminated more than 30% of embryos within this same period. Lethality and teratogenicity were not detected at concentrations below 0.63 mg/L.

### 3.2. Toxicity of ENF Alone on Zebrafish Embryo Development

**Figure 2:**
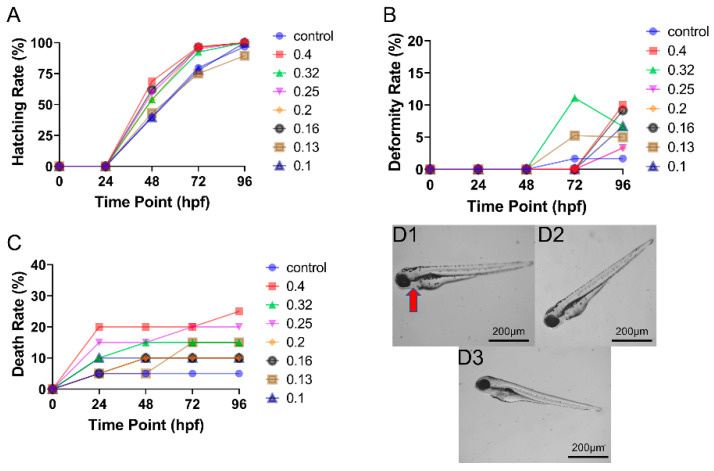
Concentration- and time-dependent effects of ENF on zebrafish embryo development (n = 60). (**A**) Time–response curves for hatching rate at all tested doses. (**B**) Time–response curves of deformity rate at all tested doses. (**C**) Time–response curves of death rate at all tested doses. (**D**) Typical deformities induced by (**D1**) 0.40 mg/L, (**D2**) 0.20 mg/L, and (**D3**) 0.10 mg/L ENF at 96 hpf. Red arrow indicates a region of pericardial oedema.

In general, ENF exhibited much lower developmental toxicity than CAR within the selected dose range (Figure 2). Enrofloxacin doses from 0.10 to 0.40 mg/L increased the hatching rate, especially from 24 to 72 hpf (Figure 2A), but with relatively modest dose dependence. Further, teratogenicity and lethality were also modest within the selected dose range, even after 72–96 hpf (Figure 2B,C), and again were not strongly dose-dependent. Indeed, at 96 hpf, there were no significant differences in deformity and death rate among concentration groups. An increase in deformity rate was not substantial until 48 to 72 hpf, while mortality increased sooner (0–24 hpf) but remained lower than CAR-induced mortality even at high doses. The deformity rate of 0.13 and 0.32 mg/L ENF at 96 hpf was lower compared to 72 hpf, which resulted from the death of deformed larvae during this period. The most common deformity in all dose groups was slight pericardial oedema (Figure 2D1).

### 3.3. Toxicity of Mixed CAR and ENF Exposure on Zebrafish Embryo Development

**Figure 3:**
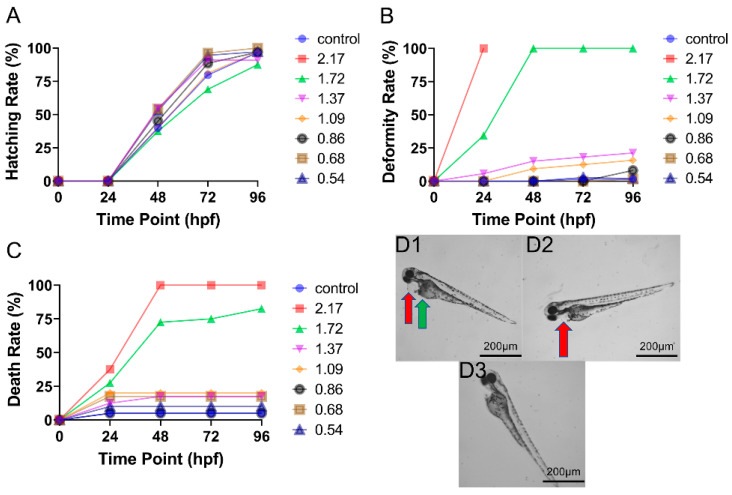
Effects of CAR plus ENF (MIX) on zebrafish development (n = 60). (**A**) Time–response curves of hatching rate at all tested doses. (**B**) Time–response curves of deformity rate at all tested doses. (**C**) Time–response curves of death rate at all tested doses. (**D**) Typical deformities induced by (**D1**) 1.72 mg/L, (**D2**) 0.86 mg/L, and (**D3**) 0.54 mg/L MIX at 96 hpf. Red arrows demarcate areas of pericardial oedema, green arrows demarcate regions of yolk sac oedema, and blue arrows indicate regions of spinal curvature.

**Figure 4:**
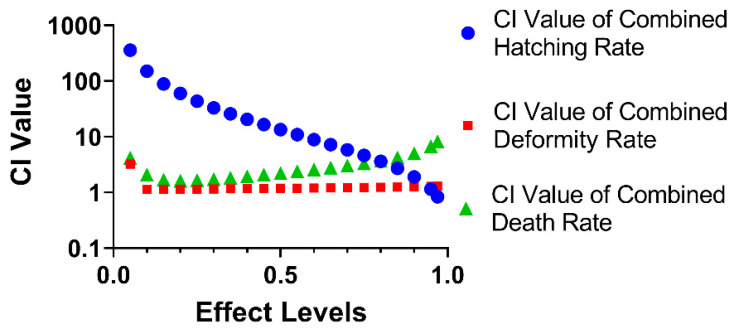
Combination indices (CI values) of MIX for 48 hpf hatching rate (blue), 96 hpf deformity rate (red), and 96 hpf death rate (green). The CI values indicate that ENF antagonized the toxic effects of CAR on hatching rate, especially at low effect levels, and on death rate at higher effect levels.

The addition of ENF (MIX treatment) reduced the developmental toxicity of CAR (Figure 3). First, the hatching rate was higher under high-dose MIX than high-dose CAR at 48 to 72 hpf (Figure 4A), while lower MIX doses had little influence on the hatching rate. Further, CAR concentrations that were teratogenic or lethal when applied alone were substantially less damaging when applied in MIX. For instance, MIX demonstrated substantial teratogenic and lethal effects only at 2.17 mg/L and 1.72 mg/L, doses containing 1.81 mg/L and 1.43 mg/L CAR, but no substantial toxicity was observed at or below 1.37 mg/L MIX, which contained a dose of CAR (1.14 mg/L) demonstrating significant teratogenicity and lethality alone. In contrast to dose-dependence, there was little effect of ENF on the time-dependence of CAR toxicity (mainly within 0–48 hpf). The inclusion of ENF also altered the specific deformities induced by CAR, as body axis curvature was rare under MIX treatment (Figure 3D1).

### 3.4. The Combined Effect of CAR and ENF on Developmental Parameters

These qualitative changes in 48 hpf hatching rate, 96 hpf deformity rate, and 96 hpf death rate between CAR alone and CAR in the presence of ENF (MIX) were then examined quantitatively by calculating combination indices (CI values), where a CI value > 1.1 indicates antagonism and a CI value less than 0.9 indicates synergism. As shown in Figure 4, ENF antagonized the effects of CAR on the hatching rate at 48 hpf, deformity rate at 96 hpf, and death rate at 96 hpf over most of the effect range, with synergism detected only in the 97–100% effect range for the 48 hpf hatching rate.

### 3.5. Transcriptomics and qPCR Analysis of Toxicity Pathways 

**Figure 5:**
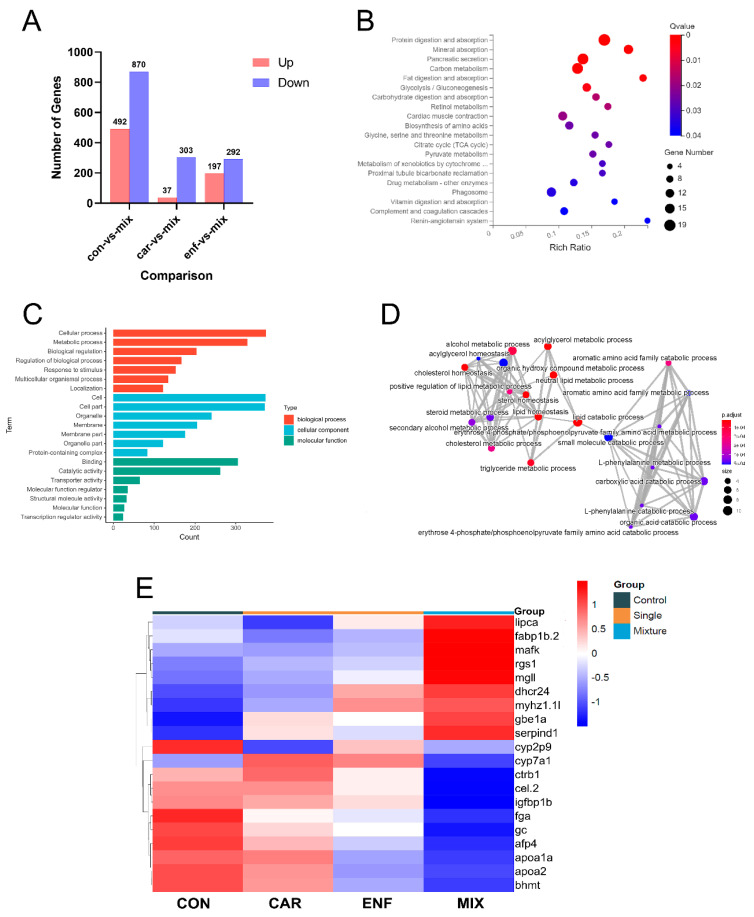
Transcriptomic results indicate that DEGs among treatment groups are enriched in genes required for nutrient metabolism. (**A**) DEGs for controls vs. MIX, CAR vs. MIX, and ENF vs. MIX at 96 hpf. (**B**) KEGG pathway enrichment of DEGs. The y-axis represents the significantly enriched KEGG pathways and the x-axis denotes the enrichment factor of DEGs. (**C**) GO category pattern of DEGs in 96 hpf larvae. (**D**) GO network of DEGs. (**E**) Heat map of top DEGs.

Since ENF mainly exhibited antagonistic effects on CAR-induced developmental toxicity, transcriptomics analysis focused on differentially expressed genes (DEGs) between MIX and single compound treatment groups. In eggs treated with MIX, 37 genes were significantly upregulated and 303 downregulated compared to the CAR treatment group (Figure 5A,B2). Compared to ENF-treated eggs, MIX treatment upregulated 197 genes and downregulated 292 genes (Figure 5A,B1). The Kyoto Encyclopedia of Genes and Genomes (KEGG) pathway analysis revealed that this DEG set was enriched in genes involved in ‘protein digestion and absorption’, ‘mineral absorption’, ‘carbon metabolism’, ‘fat metabolism’, and ‘other pathways’ (Figure 5C), while GO enrichment identified multiple genes implicated in the ‘cellular process’, ‘metabolic process’, and ‘biological regulation’ (Figure 5D). Construction of a gene network based on GO enrichment revealed that multiple DEGs were involved in ‘lipid catabolism’ and ‘small molecule metabolism’ (Figure 5E). Thus, many of these DEGs regulate the metabolism of common nutrients.

**Figure 6:**
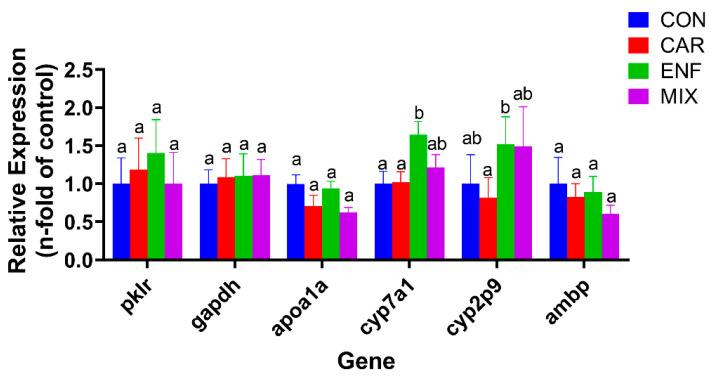
Relative mRNA expression levels of DEGs related to metabolism. Results are expressed as the fold-change relative to the control group. Data are shown as mean ± SEM; (Two-way ANOVA, Tukey’s HSD). Completely different lowercase letters above the bars indicate significant differences (*p* < 0.05), while any of the same lowercase letters indicate no significant difference (*p* > 0.05).

The heat map and qPCR results showed that CAR and ENF have distinct effects on the expression levels of many metabolism-related genes (Figure 5F and Figure 6). Specifically, both CAR and ENF promoted the expression of the metabolism-related gene *apoa1a*, while no significant changes could be found in MIX. Besides, CAR and ENF showed different impacts on the *apoa1a* gene. In addition, CAR and ENF showed antagonistic effects on expression levels of cytochrome (cyp) family genes *cyp2p9* and *cyp7a1*. Both compounds slightly increased the expression of *cyp7a1* when applied alone, but not when applied together. In contrast, MIX reduced the expression of *cyp2p9*, while neither compound alone influenced expression.

## 4. Discussion

Carbendazim residues have been detected in fruits, vegetables, and fresh grains. For example, in Loquat, Lebanon, carbendazim was detected at a maximum concentration of 0.096 mg/kg [29]. Further, the application of carbendazim to rapeseed flowers resulted in residual accumulation in the honey up to 0.35 mg/kg [30]. Carbendazim has also been detected at 0.010–0.364 mg/kg in some green tea samples from China [31]. The current study indicated that even 0.50 mg/L carbendazim can be teratogenic and lethal to zebrafish embryos. While there is no precise method to convert the residual amount in fresh foods to direct exposure of zebrafish, the recent increase in food safety research using zebrafish [32] and the strong correlation in toxicity parameters with rodents (logR = 0.7340320) [33] does allow for a simple estimation of dose equivalence. Based on this simple calculation, 0.50 mg/L is equivalent to the rodent toxicity threshold of 9.60 mg/L, which yields a theoretical ADI of 0.0096 mg/kg bw/day, lower than the existing ADI (0.03 mg/kg bw/day) [34]. Thus, the actual risk of carbendazim exposure may be higher than currently believed.

Although restrictions on the administration of ENF and other antibiotics to livestock have been effective, there are still reports of excessive residue in food. For example, reported ENF residues in pork samples from selected EU countries ranged from 0.05–2.46 μg/kg [35]. However, average levels detected in chicken breasts from Brazil ranged from 8.63 to 12.25 μg/kg [36]. These residual levels are above the ADI (allowable daily intake) evaluated by WHO (2.3 µg/kg bw [37]), but below the range causing detectable toxicity in zebrafish, the substantial health risks of ENF still need attention.

Previous studies on joint CAR toxicity have focused primarily on other pesticides [38,39] and similar studies on ENF have focused on other veterinary drugs [40,41], while few studies have examined the combination of pesticides and veterinary drugs. However, as most people consume fruits, vegetables, milk, and meat every day, an examination of potential combined effects of pesticides and veterinary drugs is important for comprehensive safety assessment. Although CAR and ENF mainly exhibit antagonistic effects at the dose ranges and ratio selected, this does not mean that ENF will mitigate the health risks of CAR as mixtures can exhibit distinct combined effects (e.g., antagonism, synergy, additivity) across concentration ranges and ratios [42,43]. Such results also highlight the importance of choosing dose ranges and ratios reflective of real-world exposure for risk assessment.

Transcriptomics and qPCR identified several hundred genes differentially expressed between treatment groups, while GO and KEGG analyses indicated that many of these DEGs are involved in the absorption or utilization of nutrients. These findings are consistent with previous reports that CAR can act as a metabolic disruptor. For instance, Bao et al. found that CAR can impair glucose and lipid metabolism in the liver of adult zebrafish [44], while others have found that CAR can impair glucose, glycerol triester, and protein regulation in mice [45]. In contrast, there are no similar reports on the metabolic effects of enrofloxacin. The longer-term effects of combined exposure on metabolism warrant further study. 

Genes differentially expressed between treatment groups included *cyp7a1*, *apoa1a*, and *pklr*. The protein product of *cyp7a1*, cytochrome P450 family 7 subfamily A member 1 (CYP7A1), is the rate-limiting enzyme for bile acid synthesis in the liver. Inhibition of *cyp7a1* may be related to tissue-specific farnesoid X receptor (Fxr) activation [46]. CYP7A1 may also be involved in the anti-hypercholesterolemia effect of plant extracts [47] and in the hepatotoxicity caused by certain anti-tuberculosis drugs [48]. Thus, abnormal expression of *cyp7a1* induced by CAR or CAR plus ENF may lead to liver damage and impaired bile acid metabolism. Apoa1a (apolipoprotein A-Ia) belongs to the apolipoprotein A family and is closely related to fat metabolism and utilization. Studies have shown that the expression of *apoa1* gene is affected by the acute toxicity of 3,4-dichloroaniline on zebrafish juveniles [49]. The affected expression of *apoa1* was also discovered in perfluorooctanesulfonic-acid-caused abnormal lipid metabolism in adult zebrafish and offspring [50]. Like *apoa1a*, the product of *pklr*, pyruvate kinase L/R (PKLR), is a glycolytic enzyme implicated in the toxicity of several other compounds. For example, the changes in HepG2 metabolism caused by bisphenol analogs were related to altered *pklr* expression [51]. Additionally, liver damage in Chinese rare minnows caused by carbamazepine may be related to altered pklr transcription [52]. Thus, CAR may fundamentally disrupt the glucose metabolism by altering the expression levels and enzymatic activities of APOA1A and PKLR. Again, the longer-term effects of CAR and MIX on glycolysis warrant further study.

## 5. Conclusions

This study demonstrated that CAR and ENF reveal toxicity on the developmental parameters of zebrafish embryos when worked alone. However, ENF can antagonize the developmental toxicity of CAR in zebrafish embryos at a ratio of 1:4.82, possibly by reciprocal effects on the expression levels of metabolic enzymes, including glycolytic enzymes. The results indicate that the cross-category toxicity of different chemicals is complex and needs more research. Given the potential for combined exposure to pesticides and veterinary drugs, it is critical to document joint toxicity at realistic doses for health risk assessment.

## Data Availability

The transcriptome raw data have been submitted to NCBI database with the BioProject number PRJNA780808.

## Supplementary Materials

The following are available online at https://www.mdpi.com/article/10.3390/toxics9120349/s1, 1. mRNA Library Construction, 2. RNA-Seq Data Analyze, Supplementary Table S1. Primer pairs of selected genes in qRT-PCR analysis.
