# Supplementary material for: Antagonistic Effects of Enrofloxacin on Carbendazim-Induced Developmental Toxicity in Zebrafish Embryos
Source: Toxics. 2021 Dec 10;9(12):349. doi: 10.3390/toxics9120349 (PMC8704853; doi:10.3390/toxics9120349)
Supplement: Supplementary file 1 [file toxics-09-00349-s001.zip › toxics-1452817-supplementary.pdf]

toxics

MDPI

Supplementary Materials: Antagonistic Effects of Enrofloxacin on Carbendazim-Induced Developmental Toxicity in Zebrafish Embryos

Ruiqi Fan, Wanjun Zhang, Li Jia, Sunlin Luo, Ying Liu, Yongpeng Jin, Yongchen Li, Xiaoyan Yuan and Yiqiang Chen

Table S1. Primer pairs of selected genes in qRT-PCR analysis.

Gene	Forward Primer	Reverse Primer
β-actin	TCAGTGCACGCTGAGAAGAT	ATGCCAACCATCACTCCCTGA
pklr	GGACTCATTCATCCAGAAGCAG	GCGATGTTCATTCCTGCTTT
gapdh	TTTGACGCTGGTGCTGGTAT	CGTATCAAAAAAAAGAAACAGCAA
apoa1a	TATGCCCAGACCACCTCCC	GGCGGTTCAGAGCGGAGT
cyp7a1	CAGGAATACGCAGAAGACATCC	TTGCCCCAAACTGAAGAGCA
cyp2p9	GTATCCTGGGATTCAAGCTAAAGTT	TATCCTCTGAAGTAGTCCTGGTCAC
ambp	GTACGCTCTGGTGGTGATGTTA	CCGCTGAATCTGTGGCTCT
